# “Because Somebody Cared about Me. That's How It Changed Things”: Homeless, Chronically Ill Patients’ Perspectives on Case Management

**DOI:** 10.1371/journal.pone.0045980

**Published:** 2012-09-28

**Authors:** Elizabeth Davis, Aracely Tamayo, Alicia Fernandez

**Affiliations:** 1 University of California San Francisco, Division of General Internal Medicine, San Francisco General Hospital, San Francisco, California, United States of America; 2 University of California Berkeley School of Public Health, Department of Epidemiology, Berkeley, California, United States of America; University of Edinburgh, United Kingdom

## Abstract

**Background:**

Case management programs for chronically ill, homeless people improve health and resource utilization by linking patients with case managers focused on improving management of medical and psychosocial problems. Little is known about participants’ perspectives on case management interventions.

**Methods:**

This qualitative study used in-depth, one-on-one interviews to understand the impact of a case management program from the perspective of participants. A standardized interview guide with open-ended questions explored experiences with the case management program and feelings about readiness to leave the program.

**Results:**

Four recurrent themes emerged: (1) Participants described profound social isolation prior to case management program enrollment; (2) Participants perceived that caring personal relationships with case managers were key to the program; (3) Participants valued assistance with navigating medical and social systems; and (4) Participants perceived that their health improved through both the interpersonal and the practical aspects of case management.

**Conclusions:**

Chronically ill, homeless people enrolled in a case management program perceived that social support from case managers resulted in improved health. Programs for this population should consider explicitly including comprehensive social support interventions. Further research on case management should explore the impact of different types of social support on outcomes for homeless chronically ill patients.

## Introduction

Chronically ill, homeless people experience poor health and adverse outcomes at a higher rate than the general population, resulting in elevated morbidity and mortality with a life expectancy about 30 years less than the US average[1]–[3] The health decline of chronically ill, homeless people is often accompanied by high rates of emergency department visits and hospitalizations, often preventable, which combined with poor use of primary care, results in costly and inefficient use of health care system resources.[4]–[15].

Case management programs for chronically ill, homeless people usually seek to improve health and avoid preventable emergency department visits and hospitalizations. Case management programs have been used in the care of the mentally ill for decades and more recently have become widely used for patients with complex medical health care needs.[16]–[18] While case management programs may differ in emphasis and approach, they tend to share common features of structured patient assessment, coordination of care, patient education, and clinical monitoring over time. [19] Case management programs specific to chronically ill, homeless populations also focus on obtaining housing and financial entitlements for clients but vary greatly in their operational characteristics such as duration, intensity and focus of services. [20] Common variants include case manager/client ratio, service focus, and case management program funding source. [20] In general, case management programs for chronically ill, homeless people appear to succeed in decreasing health service use and improving medical outcomes. Specifically, programs have been found to lower total public costs,[21]–[23] hospital days, [24] emergency department visits, and inpatient visits,[22], [23], [25]–[27] while increasing CD4 counts, [28] improving human immunodeficiency virus (HIV) viral loads, [25] and raising survival among HIV patients. [25].

Studies to date have not evaluated case management programs for chronically ill, homeless people from the perspectives of participants. Participant perceptions are important as they may shed light on program characteristics that encourage long-term participation and help distinguish core components of successful programs. This information could inform the design and improvement of case management programs. We studied the perspectives of enrollees in an intensive case management program focused on decreasing admissions among frequently admitted patients at a public hospital in order to understand: (1) what value the enrollees’ found in ongoing engagement with the program; and (2) which of the program’s many interventions were perceived by their participant enrollees as effective in improving health.

## Methods

### Ethics Statement

The University of California San Francisco Committee on Human Research and the San Francisco General Hospital Committee on Human Research approved the study. All participants in the study provided written informed consent to participate. The research team recruited participants by sending a standardized letter to staff of the case management program explaining the study. The case management staff then explained the study individually to enrollees in the case management program. Enrollees who agreed to participate were approached by research staff who explained the study and obtained consent. During the informed consent process, potential participants were informed that they would not be disadvantaged in any way by not participating in the study, and that their place in the case management program would not be affected by participating (or not) in the study. All enrollees agreed to speak with research staff, and all who were approached by research staff consented to participate in the study. No enrollees declined to participate in the study. Participants received a $10 gift certificate to a local restaurant for their involvement in the study.

### Design

We conducted a qualitative study to investigate the perspectives of intensive case management among enrollees. We used a qualitative methodology in order to gain a nuanced understanding of participants’ experiences of case management and health.

### Sampling

Participants were enrolled in a publicly funded outpatient intensive case management program at a public urban teaching hospital affiliated with the University of California, San Francisco. The aim of the program was to decrease hospital admissions among a group of frequently admitted patients.

Criteria for entry into the case management program included three or more inpatient medical admissions to the medicine, cardiology, or family medicine services in the previous 12 months, patient willingness to participate, and no involvement in any other case management program. Although homelessness was not an inclusion criteria, the majority of enrollees were homeless or marginally housed, had a diagnosed mental illness, were engaged in substance use, and had at least one chronic medical condition.

Program staff included up to four case managers who were licensed clinical social workers, one nurse, one internist, one clerk, and one psychiatrist. Twenty five to 40 patients were in the program at any given time. Each case manager conducted home visits with each of her 10–15 clients weekly or more frequently. The program staff assisted enrollees with medical issues, such as medication refills and appointments; social issues, such as obtaining housing and entitlements; and with drug and alcohol treatment. Enrollees graduated from the program when they were housed and judged to be medically stable. The average length of enrollment in the program was 18 months, but some patients were in the program for over two years. After one year of participation in the case management program, the median number of hospitalizations per participant per year dropped from four to one.

The inclusion criterion for the study was participation in the case management program. Exclusion criteria were hospitalization at the time of the study and inability to speak English. Of the 28 enrollees in the program at the time of the study, we excluded eight from study participation: six who did not speak English and two who were hospitalized at the time of recruitment. The remaining 20 enrollees all agreed to speak with research staff about the study. After in depth interviews were completed with the first 14 participants, theoretical saturation [29]–[31] was reached and research staff did not approach the remaining enrollees.

### Data Collection

One of the researchers (ED) conducted in-depth one-on-one interviews in a private location of participants’ choice, either in their home/hotel room or in an office at the hospital. Interviews were minimally directive, avoiding the imposition of investigator assumptions and biases, and the interviewer used open-ended questions (Table 1) to explore perceived reasons for recurrent hospitalizations, experiences with the case management program, and feelings about readiness to leave the program. These questions were intended to draw out perceptions about the value of case management and its various interventions, without imposing interviewer bias. Since some of the participants had low literacy, we used easily understandable wording. Interviews lasted 30–80 minutes and continued until the participant answered all questions and believed that s/he had nothing further to tell the researcher. The variation in time was due to differences in talkativeness of the participants. All interviews were audio recorded by the interviewer and they were transcribed by a commercial transcription service. One of the researchers (ED) verified the accuracy of the transcriptions by comparing them to the audio recordings.

**Table 1:** Standard Interview Guide.

1. Please tell me about your health.
2. When you entered the High User Case Management Program, you had been hospitalized a lot. What was going on that caused you to be hospitalized a lot?
3. What about now? Have any of those things changed? What impacts your health now?
4. How does the High User Case Management Program impact on your life?
5. How does the High User Case Management Program impact on your health?
6. Tell me about a good experience you have had being in the High User Case Management Program.
7. Tell me about a negative experience you have had being in the High User Case Management Program.
8. When would it be good for you to not be in the program any longer?

### Data Handling and Analysis

We analyzed the transcriptions using the constant comparative method. [30], [32] Three investigators independently developed codes from the content of the transcripts and then the coding structure was refined through iterative comparison and discussion in order to identify conceptual segments of the data. [33] We grouped related codes into broader categories and resolved coding disagreements by consensus. Finally, the three investigators re-coded all transcripts using the final coding structure.

We followed established principles including frequent team assessment of the adequacy and comprehensiveness of the results, explicit consideration of negative cases, and delineation of a relatively narrow focus of inquiry. [31].

We used Atlas.ti, a qualitative research computer program, to facilitate organization of the data. We used participant confirmation by five participants and an audit trail documenting the data analysis processes to enhance the credibility of our findings. [34], [35] The five participants agreed with the themes we identified from the study and did not offer any revisions or changes to the themes. The final results reflect the themes that emerged from the interviews and underwent subsequent participant validation.

## Results

Study participants (Table 2) included eight men and six women between 26 to 64 years; eight were African American, four were white, one was Latino, and one was Asian. Upon entry to the program, eleven were homeless, two were in subsidized housing, and one was stably housed. Since the aim of the program was to decrease admission among frequently admitted patients, they did allow housed participants into the program, but focused on homelessness since so many participants were homeless. Thirteen had both psychiatric diagnoses and a history of substance abuse in addition to chronic medical illness.

**Table 2:** Participant Characteristics.

Housing on admissionto program	Gender	Psychiatric Diagnosis	Substance use	Race/Ethnicity	Medical Diagnoses
Subsidized housing	M	Schizophrenia	Alcohol, tobacco	African American	DM, HTN, asthma, glaucoma
Homeless	F	Depression, PTSD	Cocaine, alcohol, tobacco	African American	Sickle cell disease, HTN, asthma, DM
Subsidized housing	F	Depression, PTSD	Cannabis	African American	Asthma, migraines
Homeless	M	Depression	Alcohol, cocaine, tobacco	White	Atrial fibrillation, CHF, COPD, prostate cancer, CKD
Homeless	M	Dysthymia	Heroin, alcohol, tobacco	White	HTN, amyloidosis, ESRD, hepatitis C
Homeless	F	Depression, PTSD, personality disorder	Opiates, cocaine, alcohol	African American	Lupus, antiphospholipid syndrome, stroke
Homeless	M	Depression	Cocaine, alcohol	African American	ESRD, glaucoma, uveitis, blind, HTN, CHF
Homeless	F	Depression, personality disorder	Cocaine, tobacco	African American	ESRD, CHF, COPD, HTN, hypothyroid
Housed	F	None	None	Asian	Lupus, HTN, nephritis, DVT, hepatitis B, pancreatitis
Homeless	F	PTSD	Nicotine, amphetamines	White	COPD with pulmonary failure
Homeless	M	Depression	Heroin, alcohol	African American	AIDS, Hepatitis C, cryptococcal meningitis
Homeless	M	PTSD, depression, anxiety	Alcohol, cocaine, tobacco	White	CAD, COPD, HTN, CKD, neuropathy
Homeless	M	Depression, psychosis	Alcohol, cocaine, tobacco	Latino	Pancreatitis, PUD with recurrent GI bleed, CAD
Homeless	M	Depression, PTSD	Cannabis, amphetamines	African American	Type 1 DM, asthma

CHF- congestive heart failure, DM- diabetes mellitus, COPD- chronic obstructive pulmonary disease, CKD- chronic kidney disease, ESRD- end stage renal disease on dialysis, HTN- hypertension, PUD- peptic ulcer disease, CAD- coronary artery disease, DVT- deep vein thrombosis, PTSD- post traumatic stress disorder.

Four themes emerged from the interviews: (1) Participants described profound social isolation prior to case management program enrollment; (2) Participants perceived that strong personal relationships with case managers were a key component of the program; (3) Participants valued the role of case management in facilitating the navigation of medical and social service systems; and (4) Participants perceived their health to be improved as the result of both the interpersonal and practical aspects of case management. Quotations will be used to illustrate each of these themes. The numbers following each of the quotations indicate the participant study number.

### Profound Social Isolation Prior to Case Management Program Enrollment

A profound sense of social isolation emerged repeatedly from participants’ description of their lives:

“I don’t have any family. Don’t have any friends or anything. I’m a loner in every sense of the word. It’s just - -I don’t know why, but I think I’ve been screwed over so many times and I’m tired. So I just keep to myself, you know?” (13)

While some recounted histories of physical or sexual abuse as contributing to their isolation, others blamed themselves for not reaching out to others for help. Case managers were the primary social contact for some participants. As one participant described:

“I don't really talk, so nobody really knows, except for [name of case manager]. I haven't really asked for help. I try to do it on my own, and I guess it didn't work on my own. It's probably why I’m in the situation I am right now, because I haven't asked for help, for my problem.” (7)

Other participants related their social isolation to drug use and the street environment, where they could not establish the relationships they desired.

“Mostly all the people I know of are dope fiends and whores and stuff. Those are the kind of people I've been around, so I don't know nobody else, normal people. Seeing as they were the only kind of people I know, and I don't want to be around them, I'm kind of isolated.” (12)

Participants also identified isolation as contributing to their poor health. Some described not having anyone to help them navigate health and social systems, and others described the emotional toll of not having anyone to talk to as a factor contributing to their health.

“Blood pressure up high. You know, didn't have anybody to talk to so I stayed sick. Chest – always had a pain in my chest. When you get upset, you know, when something didn't go right – when you ain't got anybody to call, you hold that stuff on the inside, it eats you up. It eats you up.”(8)

### Strong Interpersonal Relationships with Case Mangers were a Key Component of the Program

Interwoven with their descriptions of social isolation, participants explained that the gain of a personal relationship with a case manager constituted a major component of what they valued in the case management program. Participants repeatedly described feeling less isolated, more understood, and more accompanied. In speaking about his case manager, a participant explained:

“One person I’ve got to talk to is [name of case manager]…. She is a caring person. You think nobody cares for you, but somebody still does.”(12)

Participants routinely used analogies of kinship *“like a sister or an aunt”* or friendship, *“like a friend to me”,* when describing the nature of their relationships with case managers, and often expressed feeling *“cared about”*. Many described an essential aspect of the case manager as *“being there”*:

“They’re just like, just being there when nobody else is there … just there for you.”(1)

In describing their relationships with their case managers, many participants emphasized the importance of being able to speak comfortably about their illness, addiction, and homelessness. They recounted decreased feelings of shame as they developed relationships with their case managers, who they perceived as non-judgmental and caring. Feeling cared about, despite recognition of their drug and other problems, was a significant driver of positive change for the participants.

“It's helped me feel more comfortable with myself, not worrying that I'm all that bad or something. I'm still a human being that might have other problems that need to be looked at and taken care of. I used to feel like I had to hide it, hide something, or not be willing to talk to anybody about it. Just kind of, I'm going to do what I want to do and nobody else has to know of it or care about it. So now I feel a little more at ease with myself, talking to people about certain things, and just being more aware of what's going on, medically.” (5)

“So for me to be involved in this program is a very important thing in my life and I count on it, and I can count on it. I’ve gotten to know [name of case manager] really well, and we have no secrets between us. It’s like she knows that I’ve been off heroin now for two years because of this program, and I was able to do that through her counseling and being able to talk to her about certain topics where I can be really up front with her.” (6)

“Because somebody cared about me. That's how it changed things. When somebody cares about you … that makes you feel good.” (4)

### Participants Valued the Role of Case Management in Facilitating the Navigation of Medical and Social Service Systems

Participants further valued having case managers as advocates who helped them navigate the complexities of their medical care, housing, and financial entitlements. In describing her life before being in the program, one participant said:

“There was no circle [of support]. There was no help. There were no resources. There was just me and my asthma and General Hospital. That’s it. That’s all.” (8)

Along with the delivery of medications in pill boxes and assistance in scheduling and attending appointments, participants valued case managers as advocates to help them obtain needed care from their providers and health systems who were not always perceived as sympathetic or trustworthy. They appreciated that care managers often accompanied them to medical and social service appointments to help them advocate for themselves. Some noted that prior to the program, they had not wanted to attend those appointments because they were not able to get what they needed.

“What I like is going to the doctor now. Normally when I used to go to the doctor, they'd just tell me the same old thing over. And it made me not want to go. So she [case manager] started going with me and then they started to do things. I said, oh, look at this. Big change. Big change…They gotta tell her; don't just tell him, “come back next”… But soon as [name of case manager] got on it, going to the doctor with me, all of a sudden surgery [happened] just like that (snap).” (4)

“It’s like insurance, [for] when things in my health occur. I’ve had some infections get in my skin that’s led to a temperature that just knocks me out. … and these guys [case managers] smooth the way for me to get declotted or get antibiotics and I just find it extremely helpful for me.” (6)

Facilitating housing and entitlements were very important to participants. Many had tried to get housing or Social Security disability income before entering the program, but had been unable to overcome the barriers they encountered, such as abusive landlords or inappropriate denials of services.

“They helped me get on Social Security, helped me get my birth certificate, which was the hardest thing in the world for me to do by myself, to even get an ID. To just get one ID.” (4)

“So I had a room … but it was a community… we had 12 grown chickens, 12 baby chickens, a cat, a dog, and a rabbit. And I'm like, “I can't live like this.” So [name of case manager] came out for a home visit. She seen all the animals and she said, ‘God, you’ve got to get out of here. This is what’s killing you.’ And she started looking and she … showed me a picture of this place [studio apartment]. It wasn't even built yet. It was being constructed. And I told her, “I'll take it. Anything to get out of here. I'll take it!” (9)

### Improved Health was Perceived as the Result of both the Interpersonal and Practical Aspects of Case Management

Participants perceived that both the personal (feeling cared about and understood) and practical (help with medications, appointments, social services) aspects of the case management program led to improvements in their health. While participants differentiated the emotional components from the practical components of the case management program, they usually intertwined the two when discussing the impact of the program on their health, and did not indicate that one was more important than the other. One participant described the regular meetings as combining an emotional and a practical need:

“Every time I see [name of case manager], I feel okay. I feel desperate sometimes to see her. To get my medication plus talk to her about how am I doing, how I feel.” (14)

Another participant answered a general query on the impact of the program as follows:

“Beautiful. A lot of help. [name of case manager] helped me get my life back together. She really did. She keeps in touch with me. She's like a friend to me, because any time I call her, she's always there. She'll come or call me back and talk to me. If there's anything I need and she can help me, so far she's been there. And if she doesn't know, she will find out for me, I bet you. … She's just there. … It brings my life together, because I probably would be strung out really bad on crack if it wasn't for her. Because of the way she talks to me about life and my dialysis. She's a very concerned person.” (9)

The one participant who did not talk about interpersonal relationships was also the only participant who was stably housed upon entry to the case management program and had social support through her work and family. Prior to enrolling in the program, she had a poor relationship with the health care system and instability of her chronic diseases. This participant emphasized the advocacy and navigational aspect of the program.

### Negative Feelings about Case Management and Readiness to Leave the Program

Participants had very few negative feelings about case management. One participant did not like that she had to have a third party (a payee) manage her disability income for her, a requirement to gain access to housing. The same participant also did not like that her case manager played a role in limiting her opiate refills. There were no other specific negative comments.

None of the participants felt ready to leave the program. Some did not think they would ever be ready, whereas others felt they would be ready to leave when they could manage appointments and medications themselves, felt stable from a mental health perspective, and had other social contacts.

“I would never want to get out of this program… But if it came down to it, they’d have to tell me way in advance, you know what I'm saying? …Don't just come up and say, “Well, today is my last –” That would break my heart.” [8]

“If I get more or less used to making my own appointments, just more comfortable with myself, and taking any medication …Just being more at ease with myself, and more motivated to do what I'm supposed to, by coming to General, and seeing my doctor, or whatever other appointments I might have. Then I wouldn't need the program anymore…I've been saying that I just like at least having the contact, being in touch with somebody that's concerned about me…” [5]

## Discussion

### Summary of Findings

We found that participants in a case management program for frequently admitted public hospital patients identified their prior social isolation as unhealthy and valued two distinct aspects of the program: feeling cared for through their relationships with case managers and receiving assistance with navigation of medical systems and social services. Participants identified each as important contributors to their improvement.

### Examining the Findings in Light of Existing Literature

Participants linked their social isolation with their poor mental and physical health; many quantitative studies support this association. Social isolation is common among homeless people. [36] In one study of homeless people in New York City, 81% reported no weekly social contacts. [6] Decades of research have linked social isolation to poor health outcomes, including increased mortality.[37]–[43] Social isolation may be a particularly important risk factor for poor prognosis among homeless and marginalized patients, as these patients additionally have poor access to health services and are often required to navigate chaotic health care systems. [13], [44].

Berkman et al (2000) describe a framework in which social networks– the web of social contacts surrounding an individual– provide opportunities for social support, which in turn have psychological, behavioral, and physiologic impact. Social support is further broken down into emotional support (caring for others), instrumental support (providing goods and services), informational support (provision of needed information); and appraisal support (giving feedback, problem solving). [37] Using this framework, our participants described the lack of a social network (“didn’t have no one to turn to…”) and resultant lack of social support. In this context, social support provided by a care manager was filling a void left by the lack of social network. That participants valued not only instrumental and informational support, but also appraisal and emotional support can be understood in the context of their weak social network being unable to provide any of the equally important forms of social support.

### Implications for Policy, Practice, and Research

Though socially isolated patients need all forms of social support, many case management programs do not explicitly focus on emotional support. [18] This study of participants in a successful case management program demonstrates the importance of all types of social support– emotional and appraisal support as well as instrumental and informational support. While most case management programs incorporate instrumental and informational support, differing levels of focus on emotional and appraisal support could account for variance in outcomes in prior studies of case management programs. For example, if a program focuses on provision of housing (instrumental support) without focusing on caring interpersonal relationships (emotional support), that choice of focus could potentially affect outcomes. One report’s finding that person-to-person encounters and low case loads are factors associated with successful case management programs, further supports the idea that personal relationships could impact program effectiveness. [18].

How can programs operationalize this focus on emotional and appraisal support? Programs focused on patients who have no social network and who are unable to engage with peers may benefit from the very low staffing ratios and intense focus on social support demonstrated by the program in this study. While costly at the outset, this type of intervention may be what is needed to reduce preventable admissions and costs in the long run. Over time, perhaps one way to allow for graduation of patients from this type of program would be to explicitly focus on increasing social networks, thereby increasing opportunity for social support outside of the case manager relationship. This would depend on the ability of patients to be able to develop relationships with other people. In this study, although many described a lack of trust in others before being in the program (“*I’ve been screwed over so many times and I’m tired. So I just keep to myself”*), some seemed to gain self-efficacy from their relationship with their case manager (“*I feel a little more at ease with myself”*), and perhaps that self-efficacy could be leveraged to develop self sustaining social networks that could provide emotional, appraisal, informational, and instrumental support instead of case managers, similar to the model used by peer programs such as Alcoholics Anonymous.

### Strengths and Limitations

Our study has several strengths. We were able to elicit rich narratives from homeless, chronically ill participants, a population whose voices are rarely heard in health care research. Our qualitative, nonjudgmental approach allowed our participants teach us about the importance of the different types of social support. Their perspectives offer key insights into the value of case management programs. Our study also had limitations. All of the participants were English speaking and received services from the same case management program, so their experiences could be different from patients in other case management programs. Furthermore, since involvement with case management was voluntary, participants who agreed to enroll in the program may have valued health and social interaction more than frequently admitted patients who declined participation in the program. Despite our best attempts at a nonjudgmental approach, participants may still have associated the researchers with the case management program and not felt empowered to share negative feelings about the program. While our sample size was small, our use of established qualitative methods lend internal validity to our findings. Finally, we were unable to tease out which specific components of instrumental, emotional, appraisal, and informational support were most important to our participants.

### Summary

Chronically ill homeless patients account for disproportionate use of hospital resources.[13]–[15] Case management programs for these patients improve health outcomes and reduce readmissions. Participants in a case management program focused on homeless chronically ill patients perceived that social support was a key component of their success in the program. They described the importance of caring relationships with their case managers in addition to other types of social support. Case management programs should consider including mechanisms to increase social support for participants. For some populations this could include a focus on increasing social networks through peer interventions, and for other populations social support may need to come from the case manager, at least initially. Further research is needed to determine the impact of different types of social support structures within case management programs. In the meantime, case management programs for these populations should consider explicitly including emotional and appraisal support interventions. Finally, clinicians caring for chronically ill homeless patients should ask about social isolation and use available resources, such as case management or community programs, to increase social support for socially isolated patients.
